# Glutamine metabolism and its roles in tumor radiotherapy by regulating DNA damage repair

**DOI:** 10.3389/fcell.2026.1915673

**Published:** 2026-09-17

**Authors:** Yuwei Huang, Huanhuan Wang, Shuang Zhang, Zhenyang Jiang, Ruolin Shen, Weiqin Chang, Xin Jiang

**Affiliations:** 1 Jilin Provincial Key Laboratory of Radiation Oncology and Therapy, Department of Radiation Oncology, The First Hospital of Jilin University, Changchun, China; 2 Key Laboratory of Pathobiology, Ministry of Education, and College of Basic Medical Science, Jilin University, Changchun, China; 3 Healthcare Department, Affiliated Hospital of Changchun University of Traditional Chinese Medicine, Changchun, China; 4 Department of Thyroid Surgery, the Second Hospital of Jilin University, Changchun, China

**Keywords:** DNA damage response, DNA repair, double-strand breaks, genome stability, glutamine metabolism, radiotherapy

## Abstract

**Background:**

As a conditionally essential amino acid that is required by tumor cells, glutamine serves as a key metabolic hub in tumor cells, functioning both as a carbon source for the tricarboxylic acid cycle and lipid synthesis and as a nitrogen source for amino acid and nucleotide biosynthesis. Cancer cells reprogram glutamine metabolism to enhance its uptake and utilization, thereby supporting anabolic demands, maintaining redox homeostasis, and ensuring genomic stability. The efficacy of radiotherapy against malignancies largely depends on tumor cell radiosensitivity, which is influenced by DNA damage repair capacity, microenvironmental hypoxia, and cancer stem cells. Growing evidence indicates that glutamine metabolism significantly modulates tumor radiosensitivity by regulating the supply of key substrates for DNA damage repair, affecting repair protein function through post-translational modifications, and altering chromatin architecture.

**Methods:**

We performed a comprehensive literature search across PubMed and Web of Science, focusing on studies investigating the interplay among glutamine metabolism, DNA damage response, and tumor radiosensitivity. Based on critical analysis and synthesis of the retrieved literature, we propose an integrated framework linking metabolic rewiring to DNA repair modulation in the context of radiotherapy.

**Results:**

This review identifies three principal mechanisms by which glutamine metabolism may regulate the DNA damage response in tumors: (1) supplying essential substrates for DNA repair, including purines and pyrimidines derived from glutamine-dependent biosynthesis; (2) modulating the activity and function of DNA repair proteins through O-GlcNAcylation and PARylation as critical post-translational modifications, as well as regulating chromatin accessibility via histone modifications; and (3) maintaining redox homeostasis and counteracting iron-dependent lipid peroxidation to modulate ferroptosis susceptibility following irradiation. Notably, targeted inhibition of glutamine metabolism significantly enhances tumor radiosensitivity and suppresses tumor growth, demonstrating the therapeutic potential of metabolic intervention.

**Conclusion:**

Glutamine metabolism plays a multifaceted role in regulating the DNA damage response and tumor radiosensitivity. Targeting glutamine metabolic pathways, particularly through substrate supply deprivation, post-translational modification interference, or chromatin remodeling modulation, may represent a rational strategy for radiosensitization. This review provides a theoretical basis for the development of novel combination regimens integrating glutamine metabolism inhibitors with radiotherapy.

## 1 Background

Owing to their malignant proliferative properties, tumor cells exhibit significant metabolic reprogramming, a process that remodels multiple metabolic pathways to meet bioenergetic requirements, proliferative needs, and metastatic potential. Specifically, metabolic pathways such as glycolysis, glutamine catabolism, and fatty acid metabolism are enhanced to achieve efficient uptake and utilization of multiple nutrients, thereby maintaining basic cellular functions and supporting malignant phenotypes such as invasion and metastasis (Arner and Rathmell, 2023; Nguyen et al., 2025; Nabel and Vander Heiden, 2021). A central function of metabolic reprogramming, which is also a major hallmark of tumor progression and therapy resistance, is to ensure a continuous supply of essential biomolecules such as adenosine triphosphate (ATP), proteins, nucleotides, and lipids for tumor cells. This metabolic adaptation not only supports the rapid proliferation of tumor cells but also enables them to effectively respond to harsh microenvironmental stresses such as hypoxia and nutrient deprivation (Xia et al., 2021; Shu et al., 2025; Tran DH. et al., 2024; Hsu et al., 2023).

In the history of tumor metabolism research, Otto Warburg’s landmark discovery was of groundbreaking significance. Tumor cells exhibit enhanced aerobic glycolysis and maintain glucose consumption and lactate production even in a state of non-hypoxia, functions that are collectively referred to as the Warburg effect (Fendt, 2024). This unique phenomenon is now recognized as one of the core features of metabolic reprogramming in tumors (Wortley, 2022). In addition to aerobic glycolysis, tumor cells contribute to tumorigenesis, progression, and metastasis by finely regulating lipid metabolic networks (Hsu et al., 2023; Yao et al., 2023). Glutamine, together with glucose, serves as the critical metabolic substrate, and its unique redox regulatory function plays a key role in tumorigenesis and development. Recently, the phenomenon of “glutamine addiction” has received increasing attention from researchers. Many studies have shown that the growth and proliferation of tumor cells are highly dependent on a continuous supply of glutamine (Chen et al., 2023a; Wei et al., 2021). It has rendered glutamine an attractive therapeutic target, although clinical translation remains challenging. Given the important role of metabolic abnormalities in tumorigenesis and development as well as the drug resistance limitations of conventional cancer therapies, such as chemotherapy and radiotherapy, specific intervention strategies targeting tumor metabolism have become a current research focus (Stine et al., 2022).

Radiotherapy is one of the most common countermeasures used to treat cancer (Huang and Zhou, 2020), and its antitumor effects are mainly achieved through dual mechanisms. Radiation directly damages DNA by breaking its double strands through energy deposition. Moreover, radiation induces the generation of reactive oxygen species (ROS) predominantly through water radiolysis, with secondary contributions from mitochondrial electron transport chain dysfunction (Lewis et al., 2021). The cellular DNA damage response (DDR) capacity of tumor cells, particularly their DNA repair proficiency, is a key factor in determining their radiosensitivity (Kallio et al., 2025). Glutamine metabolism supports the DDR through at least two mechanisms. First, by providing essential metabolic substrates such as nucleotides for DNA repair synthesis, a relationship supported by direct experimental evidence in multiple tumor models, and second, by modulating the activity of DDR-associated enzymes through O-GlcNAcylation and PARylation, an emerging area where much of the evidence remains correlational and requires further mechanistic validation (Fu et al., 2019; Yoo et al., 2020a; Zhou et al., 2023). Preclinical evidence suggests that targeted inhibition of glutamine metabolism can impair the DNA repair ability of tumor cells and enhance oxidative stress, a strategy that has demonstrated radiosensitization effects in preclinical models of prostate, cervical, and lung cancers (Fu et al., 2019; Mukha et al., 2021a; Mukha et al., 2021b; Yao et al., 2024). It should be noted that while many of the mechanistic links between glutamine metabolism and the DDR have been established using various genotoxic agents, the extent to which these findings can be extrapolated to radiation, which induces a distinct spectrum of DNA lesions and oxidative damage, requires careful consideration and is discussed in detail herein.

The relationship between cancer metabolism and DNA damage repair has been the subject of several excellent reviews (Turgeon et al., 2018; Alden et al., 2022; Zhu et al., 2024). Building on these contributions, the present review proposes an integrated “nutrient-to-genome axis” framework that systematically connects glutamine metabolic flux to genome integrity maintenance through three mechanistically distinct but interconnected layers: (1) substrate supply for nucleotide biosynthesis (purines and pyrimidines), (2) post-translational modification of DDR proteins (O-GlcNAcylation and PARylation), and (3) chromatin remodeling and redox homeostasis regulation. Rather than examining these mechanisms in isolation, our framework emphasizes the cross-talk and feedback loops between metabolic reprogramming and DNA damage repair at multiple regulatory levels. We also provide a comprehensive analysis of how glutamine metabolism intersects with ferroptosis regulation in the context of irradiated tumor cells and a dimension that has not been systematically integrated into previous glutamine-radiosensitivity frameworks. Throughout the manuscript, we aim to critically distinguish between experimentally validated mechanisms, associative observations, and speculative hypotheses, with the goal of offering a balanced perspective that may guide future experimental and translational research.

## 2 Glutamine metabolism serves as a fundamental anabolic and bioenergetic pathway in cells

Glutamine serves as a critical hub in the cellular metabolic network, extensively participating in processes such as macromolecular biosynthesis, energy metabolism, and serving as a metabolic precursor for protein posttranslational modifications (PTMs) such as O-GlcNAcylation and PARylation (discussed in detail in Sections 4.2, 4.3). It plays a vital role in both maintaining normal cellular function and promoting cancer cell growth. However, there are significant differences in its metabolic patterns and functional regulation under physiological versus pathological conditions. This section will systematically explore the mechanisms of action and metabolic disparities of glutamine in normal versus cancer cells, aiming to elucidate its dual role in cellular homeostasis and tumor development.

### 2.1 Glutamine metabolism sustains vital functions in normal cells

Under normal physiological conditions, glutamine, a conditionally essential amino acid, is synthesized from glutamate and ammonia mainly through an ATP-dependent biosynthetic pathway catalyzed by glutamine synthetase (GS) (Lyu et al., 2024). This endogenous synthesis process is sufficient to maintain basic cellular requirements. Glutamine plays a pivotal role in cellular metabolism as a nitrogen source involved in the biosynthesis of amino acids and nucleotides, and provides a carbon skeleton for lipid synthesis (Yoo et al., 2020a; Zhu et al., 2021; Hwang et al., 2025; Liao et al., 2024; Zhang et al., 2024; Recouvreux et al., 2023). Its metabolism involves transport from the cytoplasm to the mitochondria, mediated by the SLC1A5 variant (Liu Y. et al., 2022), a mitochondrial glutamine transporter, followed by catalytic conversion to glutamate and ATP production via glutaminase isozymes (GLS1/GLS2/GAC) (Lyu et al., 2024). Subsequently, glutamate is further metabolized by glutamate dehydrogenase 1 (GLUD1/GDH1) into α-ketoglutarate (α-KG), which enters the tricarboxylic acid (TCA) cycle and provides carbon skeletons for the biosynthesis of non-essential amino acids (NEAAs), such as alanine, serine, ornithine, and aspartate, through transamination reactions (Lyu et al., 2024). In addition, glutamine, through its conversion to glutamate, serves as a precursor of glutathione (GSH) to maintain redox homeostasis and safeguard genomic stability (Tzounakas et al., 2022; Guo et al., 2020), and participates in numerous other biological processes such as chromatin modification/gene transcription, transport of other amino acids across cell membranes, and cellular signaling (Wang et al., 2018; Xu et al., 2022a).

### 2.2 Glutamine utilization is a hallmark of metabolic reprogramming in tumor

Compared with normal cells, tumor cells exhibit significant reprogramming of glutamine metabolism. To meet the energy and biomolecule synthesis requirements for their uncontrolled proliferation, cancer cells predominantly increase exogenous glutamine uptake by upregulating membrane transport proteins such as SLC1A5 and SLC38A1/A2 (Guo et al., 2023; Bröer et al., 2016), and many tumor types upregulate or maintain endogenous glutamine synthesis, creating a metabolic dependency on extracellular glutamine supply (Yang et al., 2025; Wu et al., 2026). To support the rapid proliferation of tumor cells and their need for cell membrane biosynthesis, citrate in the TCA cycle effluxes to the cytoplasm, promoting the production of acetyl coenzyme A (acetyl coA) and NADPH, a process that disrupts the integrity of the TCA cycle. As a compensatory mechanism, tumor cells significantly enhance glutamine catabolism to maintain metabolic homeostasis (Villar et al., 2015). In addition, glutamine is heavily consumed by tumor cells to maintain lipid metabolism. Glutamine also meets the tumor cells’ exuberant anabolic demands for biomolecules, such as nucleic acids, lipids, and proteins, and enhances cellular resistance to apoptosis by increasing the production of GSH (Wang et al., 2025a; Jiang W. et al., 2023). Most solid tumors, including pancreatic cancer and hepatocellular carcinoma, contain hypoxic regions that influence glutamine metabolism (Sun et al., 2023; Ding et al., 2023; Husain et al., 2022), in which they tend to enhance glutamine metabolism. In pancreatic cancer, a hypoxic microenvironment induces significantly high expression of the SLC1A5 variant, which in turn enhances glutamine catabolism and has been associated with gemcitabine resistance in preclinical studies (Yoo et al., 2020b), although further clinical validation is warranted.

Based on the aforementioned metabolic features, we systematically summarized the differences in glutamine metabolism and the metabolic pathways involved in the regulation of cellular metabolism and the synthesis of biomolecules between normal and tumor cells (Figure 1). Given the specific dependence of tumor cells on glutamine metabolism, targeted intervention in this metabolic pathway (e.g., via glutamine deprivation) has been shown to inhibit proliferation and induce apoptosis in preclinical models of various malignant tumors such as neuroblastoma and hepatocellular carcinoma (Qing et al., 2012; Chen and Cui, 2015), providing a preclinical rationale for the exploration of glutamine-targeted therapeutic strategies, though clinical translation remains an ongoing challenge. Among therapeutic modalities, radiotherapy occupies a unique position in that ionizing radiation induces both direct DNA double-strand breaks (DSBs) and secondary oxidative damage through water radiolysis, and the dual mechanism leads to a metabolic dependence that is not exactly the same as that caused by chemotherapeutic drugs. The intersection of glutamine metabolism with radiation-induced DNA damage response and redox homeostasis is discussed in detail in the following sections.

**FIGURE 1:**
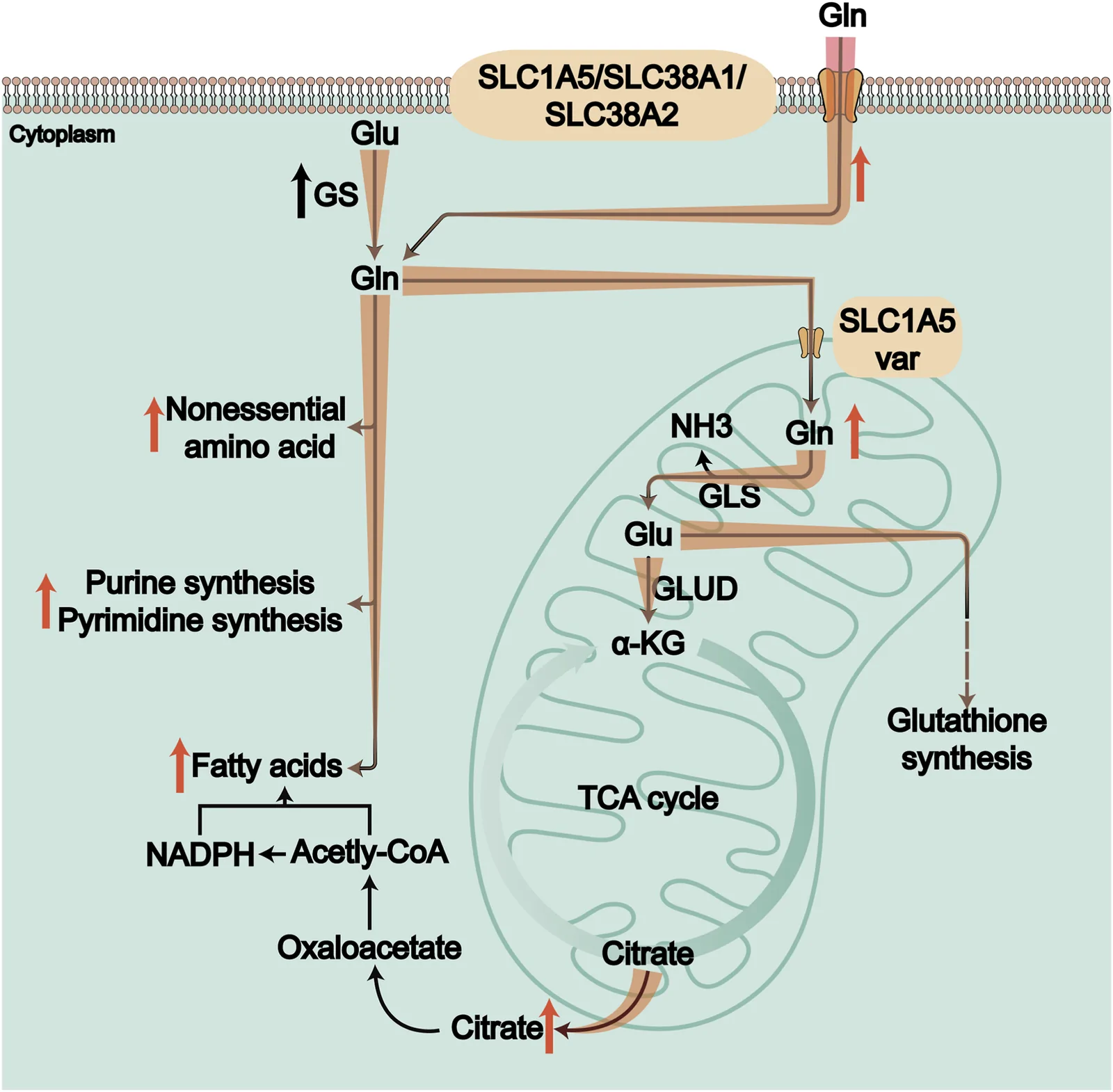
Enhanced glutamine metabolism in tumor cells. In tumor cells, glutamine (Gln) is imported from the extracellular space into the cytoplasm via upregulated plasma membrane transporters, including SLC1A5, SLC38A1, and SLC38A2. In addition to endogenous Gln synthesis catalyzed by glutamine synthetase (GS), the markedly increased exogenous Gln uptake supports multiple anabolic processes: (i) transamination-dependent biosynthesis of nonessential amino acids; (ii) *de novo* purine and pyrimidine synthesis; (iii) citrate efflux from mitochondria to the cytoplasm, providing acetyl-CoA and NADPH for fatty acid synthesis; and (iv) glutathione synthesis for redox homeostasis. Within mitochondria, the SLC1A5 variant (SLC1A5 var) transports Gln into the mitochondrial matrix, where glutaminase (GLS) converts it to glutamate (Glu) with the release of ammonia (NH_3_). Glu is subsequently deaminated to α-ketoglutarate (α-KG) by glutamate dehydrogenase (GLUD), which replenishes the tricarboxylic acid (TCA) cycle. Red upward arrows (↑) indicate metabolic pathways that are upregulated in tumor cells relative to normal cells. Black arrows indicate that GS expression varies across tumor types, remaining unchanged in some and being upregulated in others relative to normal tissues. Gln, glutamine; Glu, glutamate; GS, glutamine synthetase; α-KG, α-ketoglutarate; TCA, tricarboxylic acid; GLS, glutaminase; GLUD, glutamate dehydrogenase; SLC1A5 var, SLC1A5 variant.

## 3 DNA damage repair play a critical role in the cellular response to IR

Radiotherapy is one of the cornerstone modalities in the clinical management of malignant tumors, yet the efficacy of radiotherapy largely depends on the level of radiotherapy resistance (RR) in the tumor cells (Niu et al., 2022; Park et al., 2019). Elucidating the biological mechanisms through which radiotherapy exerts its tumoricidal effects is crucial for overcoming RR and identifying novel therapeutic targets. This section will begin with an overview of the mechanisms underlying radiotherapy, systematically analyze key processes involved in the development of RR, and subsequently explore potential new strategies and approaches to enhance therapeutic outcomes.

### 3.1 IR kills tumor cells through multiple mechanisms

Many factors affect RR, including the cancer microenvironment (Lan et al., 2021; Jeon et al., 2023), DNA repair capacity (LI H-S. et al., 2025), resistance to oxidative stress (Fan X. et al., 2025), chromatin structure (Tian et al., 2025), and cancer stem cells (CSCs) (Li et al., 2023). Radiotherapy induces cytotoxic effects on tumor cells through two main mechanisms: i) by directly depositing energy into DNA molecules, causing sugar-phosphate backbone breakage that results in single-strand breaks (SSBs) and, most critically, DSBs, as well as base damage and abasic sites, and ii) by triggering the generation of ROS to cause oxidative damage. DNA damage frequently leads to adverse events such as nuclear envelope rupture, disruption of DNA replication and transcription, and induction of apoptosis (Polo and Jackson, 2011; Huang et al., 2024; Karakostis et al., 2024). Cells exposed to radiation sustain DNA damage, which rapidly initiates a series of DDR. This multilevel regulatory process consists of three key components: (1) damage perception and transmission, (2) damage response (including DNA damage repair, cell cycle arrest, and chromosome structure alteration), and (3) the final decision of cell fate (repair or programmed death) (Claessens et al., 2023; Alagia et al., 2025; Welz et al., 2021; Chang et al., 2021; Huang et al., 2023).

DNA damage repair, the core execution phase of DDR, specializes in the identification and repair of damaged sites to maintain genome stability. Depending on the type of damage, cells can activate specific repair pathways, including base excision repair (BER) to deal with single-strand breaks; nucleotide excision repair (NER) to repair bulky helix-distorting lesions; mismatch repair to correct base pair mismatches, insertions, and deletions; and homologous recombination (HR) and nonhomologous end-joining (NHEJ) to repair DNA double-strand breaks (Liu et al., 2025; Mevissen et al., 2024; Chen Y. et al., 2023; Fan Y. et al., 2025). DDR encompasses DNA damage repair as its central execution component, while also coordinating cell cycle checkpoints, chromatin remodeling, and cell fate decisions. When repair fails, persistently activated DDR signaling drives cells to post-mitotic death, thereby preventing genomically unstable cells from continuing to proliferate (Huang et al., 2024; Rello-Varona et al., 2010). This fine-grained cascade ensures both repair efficiency and the avoidance of mutation accumulation, which are essential for maintaining tissue homeostasis. The described DNA damage and repair mechanisms are systematically summarized in Figure 2 to elucidate the resulting cell fate-determining network.

**FIGURE 2:**
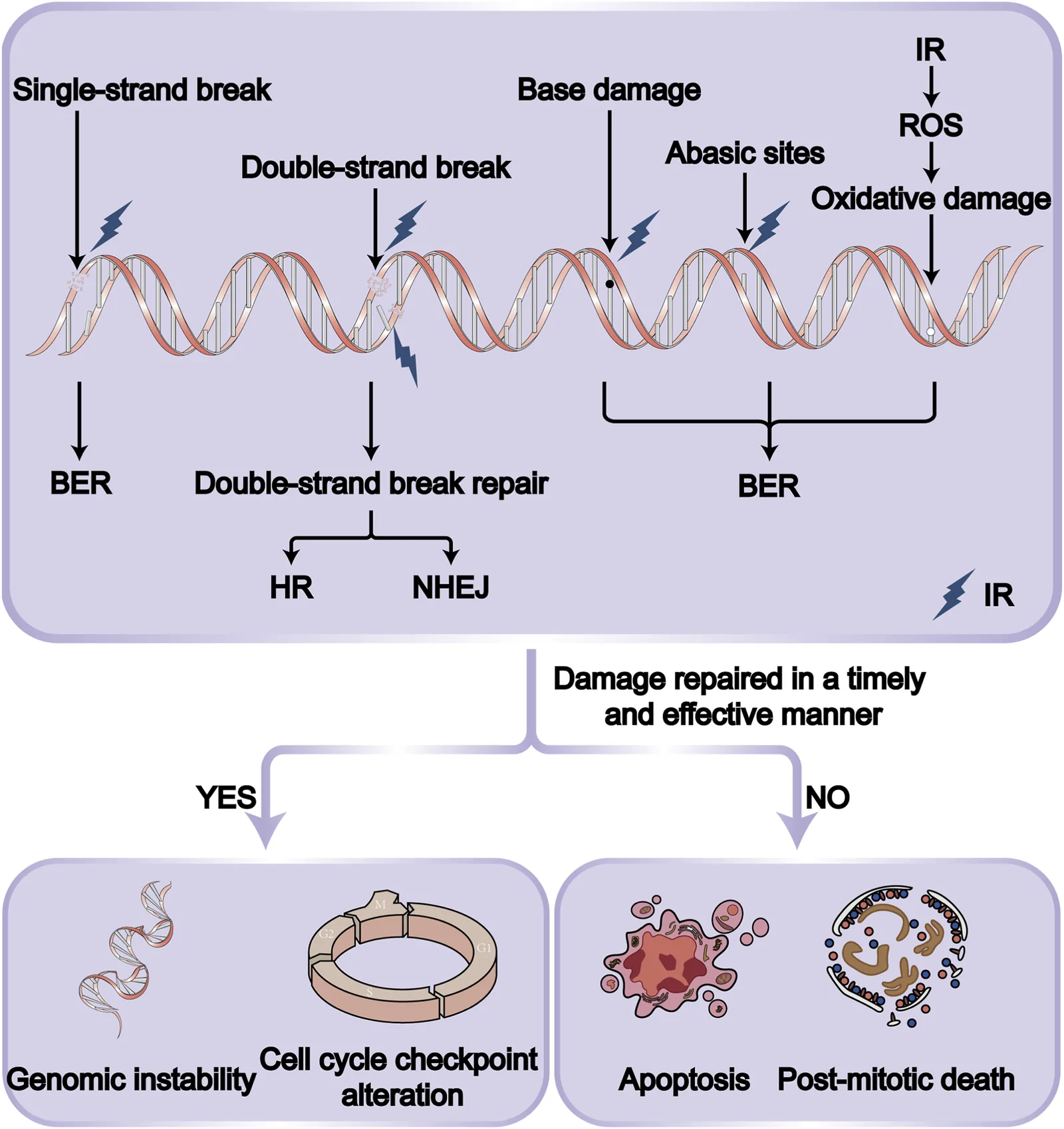
Radiation regulates cell fate by inducing DNA damage and repair. IR damages DNA through two main mechanisms: direct energy deposition causing sugar-phosphate backbone breakage, and indirect oxidative damage via water radiolysis-generated reactive oxygen species (ROS). The resulting DNA lesions include double-strand breaks (DSBs), single-strand breaks (SSBs), base damage (e.g., oxidized bases), and abasic sites. Cells respond by activating specific DNA repair pathways: base excision repair (BER) for SSBs and base lesions, and homologous recombination (HR) or non-homologous end joining (NHEJ) for DSBs. When DNA damage is repaired in a timely and effective manner, cells may survive but can still accumulate genomic instability and cell cycle checkpoint alterations. When repair fails, persistently activated DNA damage response signaling drives cells toward apoptosis or post-mitotic death, preventing the propagation of genomically unstable cells. IR, ionizing radiation; DSBs, DNA double-strand breaks; SSBs, single-strand breaks; BER, base excision repair; HR, homologous recombination; NHEJ, nonhomologous end joining; ROS, reactive oxygen species.

### 3.2 From mechanism to clinic: overcoming radioresistance and developing novel therapeutic targets

It should be noted that while numerous DDR inhibitors have demonstrated synergistic effects with various genotoxic agents, the evidence specifically supporting their role as radiosensitizers (rather than general chemosensitizers) remains largely at the preclinical stage for most agents, with only a limited number progressing to clinical trials in combination with radiotherapy (García et al., 2022; Dillon et al., 2025). This distinction is critical, as the pharmacodynamic interaction between DDR inhibition and radiation may differ qualitatively from that observed with chemotherapy, given the unique spatial and temporal patterns of radiation-induced DNA damage (Van Bijsterveldt et al., 2020; Radstake et al., 2024).

#### 3.2.1 Ataxia telangiectasia mutated (ATM)

ATM is a kinase belonging to the phosphoinositide 3-kinase-related kinase (PIKK) family (Huang et al., 2023). In cancers such as triple-negative breast cancer (TNBC), glioblastoma (GBM), and colorectal cancer (CRC), inhibition of ATM kinase activity has been shown to significantly enhance the radiosensitivity of cancer cells (Ruiz-Fernández De Córdoba et al., 2025; Chen J. et al., 2024; Xie et al., 2025). In breast cancer, inhibitors of PARP, a key DNA damage sensor, and ATM inhibitors significantly increased the radiosensitivity of tumor cells by impairing DNA damage sensing and repair signaling (Ruiz-Fernández De Córdoba et al., 2025). Corresponding small-molecule inhibitors (e.g., AZD1390, AZD0156) have demonstrated potential as radiosensitizers in preclinical studies (Chen J. et al., 2024; Xie et al., 2025).

#### 3.2.2 DNA-dependent protein kinase (DNA-PK)

DNA-PK (DNA-dependent protein kinase) is a serine/threonine kinase located in the nucleus (Menolfi and Zha, 2021), which plays a critical role in the repair of DNA double-strand breaks. It is primarily activated during the early stages of NHEJ and serves as a core regulatory protein in this repair mechanism. Studies have shown that in non-small-cell lung cancer (NSCLC) cells, the activity of DNA-PK can be blocked by the inhibitor AZD7648, thereby effectively enhancing the radiosensitivity of cancer cells (Nakamura et al., 2021).

#### 3.2.3 ATM and Rad3 related (ATR)

The ATR signaling pathway is primarily activated in response to single-stranded DNA (ssDNA), typically arising from replication stress or DNA end resection, and plays a central role in the intra-S and G2/M checkpoint activation (Drew et al., 2024; García-Rodríguez et al., 2024; Soni et al., 2022; Bright et al., 2024). Furthermore, loss of ATR function can lead to nuclear envelope rupture, resulting in genomic instability and irreparable DNA damage, which subsequently upregulates p53 and induces p53-mediated apoptosis (Wang Y. et al., 2024). In the field of cancer therapy, ATR inhibitors demonstrate anti-tumor potential through diverse mechanisms. For example, berzosertib can enhance the radiosensitivity of CRC by increasing DNA damage and alleviating radiation-induced G2/M phase arrest (Liu C. et al., 2023). Furthermore, ceralasertib shows efficacy in KRAS-mutated non-small cell lung cancer (Galan-Cobo et al., 2025), elimusertib enhances the therapeutic effect of cisplatin in hepatoblastoma (Morel-Ribeiro et al., 2025), and AZD-6738 exhibits therapeutic activity in TP53-mutated CRC (Inoue et al., 2021).

Currently, a variety of DDR inhibitors have demonstrated significant potential for clinical translation. These agents not only enhance the accumulation of DNA damage within tumor cells but also produce marked synergistic antitumor effects when combined with immune checkpoint inhibitors, chemotherapy, or radiotherapy (Ruiz-Fernández De Córdoba et al., 2025; Shen et al., 2024; Chen H. et al., 2024). Thus, inhibiting DNA damage repair to overcome tumor radiation resistance may provide a new strategy for improving the clinical outcomes of patients with cancer.

## 4 Glutamine addiction in tumor cells contributes to radioresistance through metabolic adaptation and PTMs

Glutamine addiction in tumor cells supports DNA damage repair and radioresistance through multiple interconnected mechanisms, including providing essential substrates for nucleotide synthesis, modulating the O-GlcNAcylation of DNA repair proteins via the GFAT-HBP axis, regulating PARylation-dependent chromatin remodeling, and maintaining redox homeostasis to counteract IR-induced oxidative damage.

### 4.1 Glutamine mediates nucleotide biosynthesis and DNA repair substrate availability

Glutamine serves as an essential nitrogen donor for the *de novo* biosynthesis of both purine and pyrimidine nucleotides, thereby maintaining the intracellular nucleotide pool homeostasis (Yoo et al., 2020a). This biochemical function carries critical biological consequences: following radiation therapy, efficient DNA damage repair is pivotal for the survival of tumor cells (Helleday et al., 2008). The repair process is highly dependent on the local concentration of available deoxynucleoside triphosphates (dNTPs) (Yu et al., 2025). Notably, the concept relevant to DNA damage repair is not merely the abundance of nucleotides, but rather the maintenance of adequate and balanced dNTP pools following radiation-induced damage, as both insufficient and excessive dNTP levels can impair repair fidelity (Mullen and Singh, 2023). Consequently, the nucleotide synthesis capacity supported by glutamine metabolism profoundly influences the potential of tumor cells to repair radiation-induced DNA damage and is a significant contributor to their radiosensitivity. The following sections will first elaborate on the specific mechanisms by which glutamine drives purine and pyrimidine biosynthesis, then delve into how nucleotide availability regulates the DNA damage repair process, thereby revealing the potential therapeutic value of targeting this metabolic axis to radiosensitize tumors.

#### 4.1.1 Glutamine serves as a nitrogen donor in *de novo* purine biosynthesis

*De novo* purine biosynthesis is a ten-step pathway in which glutamine serves as the amide nitrogen donor at three critical steps (Pareek et al., 2020). First, phosphoribosyl pyrophosphate amidotransferase (PPAT) catalyzes the conversion of phosphoribosyl pyrophosphate (PRPP) to phosphoribosylamine (PRA), with glutamine providing the amide nitrogen that is incorporated into the purine ring (Wu et al., 2025). Subsequently, phosphoribosylformylglycinamidine synthase (PFAS) catalyzes the fourth step, converting N2-formyl-N1-(5-phospho-d-ribosyl)glycinamide (FGAR) to 2-(formamido)-N1-(5-phospho-d-ribosyl) acetamidine (FGAM), in which a second glutamine-derived amide nitrogen is incorporated (Sharma et al., 2026). Third, guanosine monophosphate (GMP) synthetase (GMPS) catalyzes the conversion of xanthosine monophosphate (XMP) to guanosine monophosphate (GMP), utilizing glutamine as the amide nitrogen donor (Welin et al., 2013; Moffatt and Ashihara, 2002). Collectively, these three reactions consume two glutamine molecules per inosine monophosphate (IMP) molecule synthesized, one each at the PPAT and PFAS steps, with an additional glutamine required for IMP-to-GMP conversion. ^15^N-glutamine metabolic flux tracing has confirmed that the two glutamine-derived amide nitrogens are ultimately incorporated into the N3 and N9 positions of the purine ring (Tran DH. et al., 2024; Fu et al., 2019).

#### 4.1.2 Glutamine-dependent pyrimidine biosynthesis and tumor metabolic vulnerability

*De novo* pyrimidine biosynthesis commences with the multifunctional enzyme Dihydroorotase (CAD), which catalyzes the first three sequential reactions of the pathway (Ma et al., 2025; Qi et al., 2025). CAD comprises four enzymatic domains: the glutamine amidotransferase (GATase) and carbamoyl phosphate synthetase (CPS) II domains, which together catalyze the first step, followed by aspartate transcarbamylase (ATCase) and dihydroorotase (DHOase) domains, which catalyze the subsequent two steps (Del Caño-Ochoa et al., 2023). The GATase and CPSII activity of CAD cooperate to initiate pyrimidine synthesis by utilizing glutamine-derived amide nitrogen, HCO_3_^−^, and ATP to produce carbamoyl phosphate (CP) (Qi et al., 2025; Da et al., 2025). The subsequent cytosolic steps, including the condensation of CP with aspartate catalyzed by the ATCase and the cyclization of carbamoyl aspartate (C-Asp) to dihydroorotate (DHOA) by the DHOase of CAD, proceed in the cytosol, while the oxidation of DHOA to orotate (OA) by dihydroorotate dehydrogenase (DHODH) constitutes the sole mitochondrial step in *de novo* pyrimidine biosynthesis (Yang C. et al., 2023). Following orotate formation, uridine 5′-monophosphate synthase (UMPS) converts orotate to uridine monophosphate (UMP), which is subsequently phosphorylated to UDP and then UTP (Yang C. et al., 2023). UTP is aminated to cytidine triphosphate (CTP) by CTP synthase (CTPS) in an ATP-dependent, glutamine-driven reaction (Guo et al., 2025). Finally, CTP is converted to CMP through CDP, completing the biosynthetic route from orotate to UMP and CMP (Mullen and Singh, 2023). The pyrimidine ring incorporates one nitrogen from glutamine, one nitrogen and three carbons from aspartate, and one carbon from bicarbonate (Pareek and Benkovic, 2025). Notably, while aspartate directly contributes atoms to the pyrimidine ring, its carbon skeleton is largely derived from glutamine via TCA cycle intermediates (oxaloacetate) (Gorgoglione et al., 2022), establishing an indirect but metabolically significant link between glutamine metabolism and pyrimidine biosynthesis. These processes are schematically depicted in Figure 3.

**FIGURE 3:**
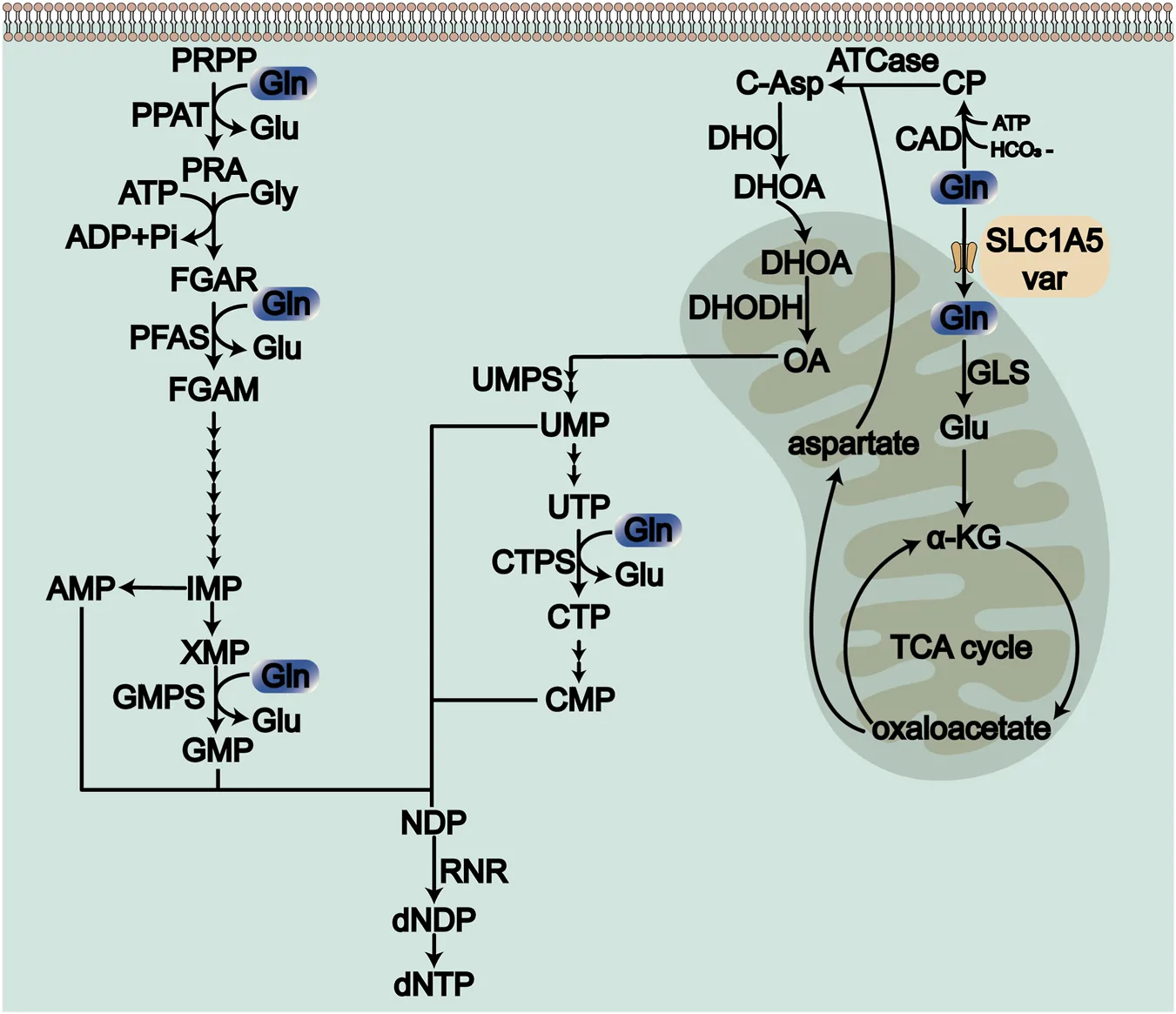
Glutamine metabolism provides the substrates of DNA repair. In tumor cells, glutamine (Gln) is imported into the cytoplasm via plasma membrane transporters and into mitochondria via the SLC1A5 variant (SLC1A5 var). Within mitochondria, glutaminase (GLS) converts Gln to glutamate (Glu), which is further metabolized to α-ketoglutarate (α-KG) and enters the tricarboxylic acid (TCA) cycle, generating oxaloacetate and subsequently aspartate—a key carbon and nitrogen donor for pyrimidine biosynthesis. For purine biosynthesis, glutamine serves as the amide nitrogen donor at three steps: phosphoribosyl pyrophosphate amidotransferase (PPAT) catalyzes the first committed step, phosphoribosylformylglycinamidine synthase (PFAS) catalyzes the fourth step, and GMP synthetase (GMPS) catalyzes the conversion of XMP to GMP. For pyrimidine biosynthesis, the multifunctional enzyme CAD catalyzes the first three steps, utilizing glutamine-derived amide nitrogen to produce carbamoyl phosphate (CP); aspartate transcarbamylase (ATCase) then condenses CP with aspartate to form carbamoyl aspartate (C-Asp); and dihydroorotase (DHOase) cyclizes C-Asp to dihydroorotate (DHOA). Dihydroorotate dehydrogenase (DHODH) oxidizes DHOA to orotate (OA), and uridine 5′-monophosphate synthase (UMPS) converts OA to UMP, which is phosphorylated to UTP. CTP synthase (CTPS) then aminates UTP to CTP using glutamine as the nitrogen donor. Ribonucleotides are converted to deoxynucleotides (dNTPs) by ribonucleotide reductase (RNR) to provide the direct substrates for DNA repair synthesis. Gln, glutamine; Glu, glutamate; α-KG, α-ketoglutarate; GLS, glutaminase; SLC1A5 var, mitochondrial glutamine transporter variant; TCA, tricarboxylic acid; PRPP, 5-phosphoribosyl-1-pyrophosphate; PPAT, phosphoribosyl pyrophosphate amidotransferase; PRA, phosphoribosylamine; ATP, adenosine triphosphate; ADP, adenosine diphosphate; Gly, glycine; FGAR, N2-formyl-N1-(5-phospho-D-ribosyl)glycinamide; PFAS, phosphoribosylformylglycinamidine synthase; FGAM, 2-(formamido)-N1-(5-phospho-D-ribosyl)acetamidine; IMP, inosine monophosphate; XMP, xanthosine monophosphate; GMPS, GMP synthetase; GMP, guanosine monophosphate; AMP, adenosine monophosphate; CP, carbamoyl phosphate; ATCase, aspartate transcarbamylase (CAD domain); C-Asp, carbamoyl aspartate; DHOA, dihydroorotate; DHODH, dihydroorotate dehydrogenase; OA, orotate; UMPS, uridine 5′-monophosphate synthase; UMP, uridine monophosphate; UDP, uridine diphosphate; UTP, uridine triphosphate; CTPS, CTP synthase; CTP, cytidine triphosphate; CMP, cytidine monophosphate; RNR, ribonucleotide reductase; dNTP, deoxynucleoside triphosphate.

Experimental evidence has demonstrated that glutamine-dependent pyrimidine synthesis is linked to cancer metabolic vulnerabilities (Cardenas et al., 2026; Liu F. et al., 2022). Inhibition of CAD or depletion of intracellular glutamine leads to pyrimidine exhaustion, cell cycle arrest, and impaired DNA replication (Qi et al., 2025; Shin et al., 2023). While direct evidence specifically linking glutamine-dependent pyrimidine biosynthesis to radiotherapy outcomes remains limited, the general requirement for adequate pyrimidine pools in DNA damage repair suggests that dNTP scarcity could compromise both DNA synthesis-dependent repair pathways and replication fork stability following IR-induced damage (TENG et al., 2022; Das et al., 2023; Pandey et al., 2025).

#### 4.1.3 dNTP pool homeostasis and DNA damage repair efficiency

*De novo* purine and pyrimidine biosynthesis initially generates ribonucleotides (ATP, GTP, CTP, UTP) (Mullen and Singh, 2023). Their conversion to dNTPs, the substrates required for DNA replication and repair, is catalyzed by ribonucleotide reductase (RNR), a rate-limiting enzyme tightly regulated by allosteric mechanisms to maintain balanced dNTP pools (Awoyomi et al., 2025; Tran P. et al., 2024). Both insufficient dNTP levels, which cause replication stress and repair failure, and excessive dNTP imbalances, which drive mutagenesis, can impair repair fidelity (Tran P. et al., 2024; Shu et al., 2020). A growing body of evidence indicates that glutamine-supported nucleotide biosynthesis directly enhances DNA repair capacity (Mullen and Singh, 2023; Shin et al., 2023; Aird et al., 2015). In IMR90 human fibroblasts and CNE2 radiation-resistant nasopharyngeal carcinoma cells, enhanced glutamine uptake significantly elevated intracellular dNTP pools and reduced DSBs following ionizing radiation (Fu et al., 2019; Aird et al., 2015). Consistent findings have been reported in breast cancer models, where glutamine reduced irradiation-induced DNA damage (Cano et al., 2010) and promoted DSB repair (Mullen and Singh, 2023; Efimova et al., 2016). Conversely, inhibition of glutamine metabolism leads to nucleotide depletion, compromising both the kinetics and pathway selection of DNA damage repair (Kery and Papandreou, 2020).

Notably, tumor cells can circumvent impaired *de novo* pyrimidine synthesis by activating the salvage pathway, utilizing extracellular nucleosides and nucleobases to maintain nucleotide pool homeostasis (Sexauer et al., 2023; Zhao J. et al., 2025). Extracellular nucleosides, particularly uridine, are imported via nucleoside transporters and phosphorylated by salvage enzymes such as uridine-cytidine kinase (UCK) to replenish the pyrimidine pool in a DHODH-independent manner (Pham et al., 2025; Mullen et al., 2022). Consistently, exogenous uridine supplementation has been shown to rescue cancer cells from DHODH inhibition, underscoring the clinical relevance of this compensatory mechanism and the need for combination strategies targeting both *de novo* and salvage pathways (Mullen et al., 2022; Tarullo et al., 2024). Whether such salvage-mediated rescue similarly protects tumor cells from radiation-induced damage remains to be determined. These findings suggest that modulating nucleotide metabolism to enhance post-radiotherapy DNA damage accumulation may represent a potential radiosensitization strategy, though direct clinical evidence remains to be established (Pranzini et al., 2022; Yu et al., 2023).

### 4.2 Glutamine regulates the O-GlcNAcylation of proteins associated with DNA damage repair

Research has shown that the biological activities of DNA damage repair enzymes and chromatin-associated proteins, key effector molecules of the DNA damage repair system, are finely regulated by a variety of PTMs. These reversible modifications include, but are not limited to, SUMOylation, ubiquitylation, O-linked β-N-acetylglucosamine modification (O-GlcNAcylation) and poly-ADP-ribosylation (PARylation), which comprise a complex regulatory network for DNA damage repair by altering protein conformation, subcellular localization, or protein-protein interactions (Zhou et al., 2023; Zhao H. et al., 2025; Becker et al., 2021; Zhao et al., 2022). Glutamine availability can modulate O-GlcNAcylation levels by fueling the hexosamine biosynthetic pathway (HBP), which generates the essential substrate, uridine 5′-diphosphate (UDP)-N-acetylglucosamine (UDP-GlcNAc). Therefore, subsequent chapters will systematically analyze the regulatory network connecting glutamine metabolism, O-GlcNAcylation, and DNA damage repair, aiming to elucidate the mechanisms through which cellular metabolism influences genomic stability.

#### 4.2.1 Glutamine drives O-GlcNAcylation via the GFAT-HBP axis

O-GlcNAcylation is a unique and dynamic form of PTMs that involves the covalent attachment of O-linked β-N-acetylglucosamine (O-GlcNAc) sugar molecules to the hydroxyl groups of serine or threonine residues in the target proteins (Zhou et al., 2023). Unlike classical N-linked and O-linked glycosylation, which occurs in the endoplasmic reticulum and Golgi apparatus to generate complex glycoproteins destined for secretion or membrane insertion, O-GlcNAcylation is primarily localized to nuclear and cytosolic compartments (Zhang and Wang, 2024; Formichetti et al., 2025). In contrast to classical glycosylation, which generates complex and branched oligosaccharide chains through hundreds of glycosyltransferases using diverse nucleotide sugar donors, O-GlcNAcylation employs a single nucleotide sugar donor, UDP-GlcNAc (Yan et al., 2025). This modification process is finely regulated by two key enzymes: O-linked N-acetyl-glucosaminyltransferase (OGT), which is responsible for catalyzing the addition of O-GlcNAc, and O-linked N-acetyl β-D-glucosaminidase (OGA), which mediates its removal, together establishing a highly dynamic modification cycle that is sensitive to fluctuations in cellular UDP-GlcNAc levels (Yang Y. et al., 2023).

The metabolic connection between glutamine and O-GlcNAcylation is established through HBP (Zhou et al., 2025; Su et al., 2025). Glutamine-fructose-6-phosphate amidotransferase (GFAT), the rate-limiting enzyme of the HBP, catalyzes the transfer of the amide nitrogen from glutamine to fructose-6-phosphate (Fru-6-P) to generate glucosamine-6-phosphate (GlcN-6-P), thereby linking intracellular glutamine availability to UDP-GlcNAc biosynthesis (Zhou et al., 2025). Consequently, reduced glutamine levels significantly diminish UDP-GlcNAc production and global O-GlcNAcylation (Zhou et al., 2023; Swamy et al., 2016). It should be emphasized that this review specifically addresses OGT-mediated O-GlcNAcylation of nuclear and cytosolic DDR proteins, rather than classical secretory-pathway glycosylation. The functional implications of this modification for DNA damage repair are discussed in the following section (Figure 4).

**FIGURE 4:**
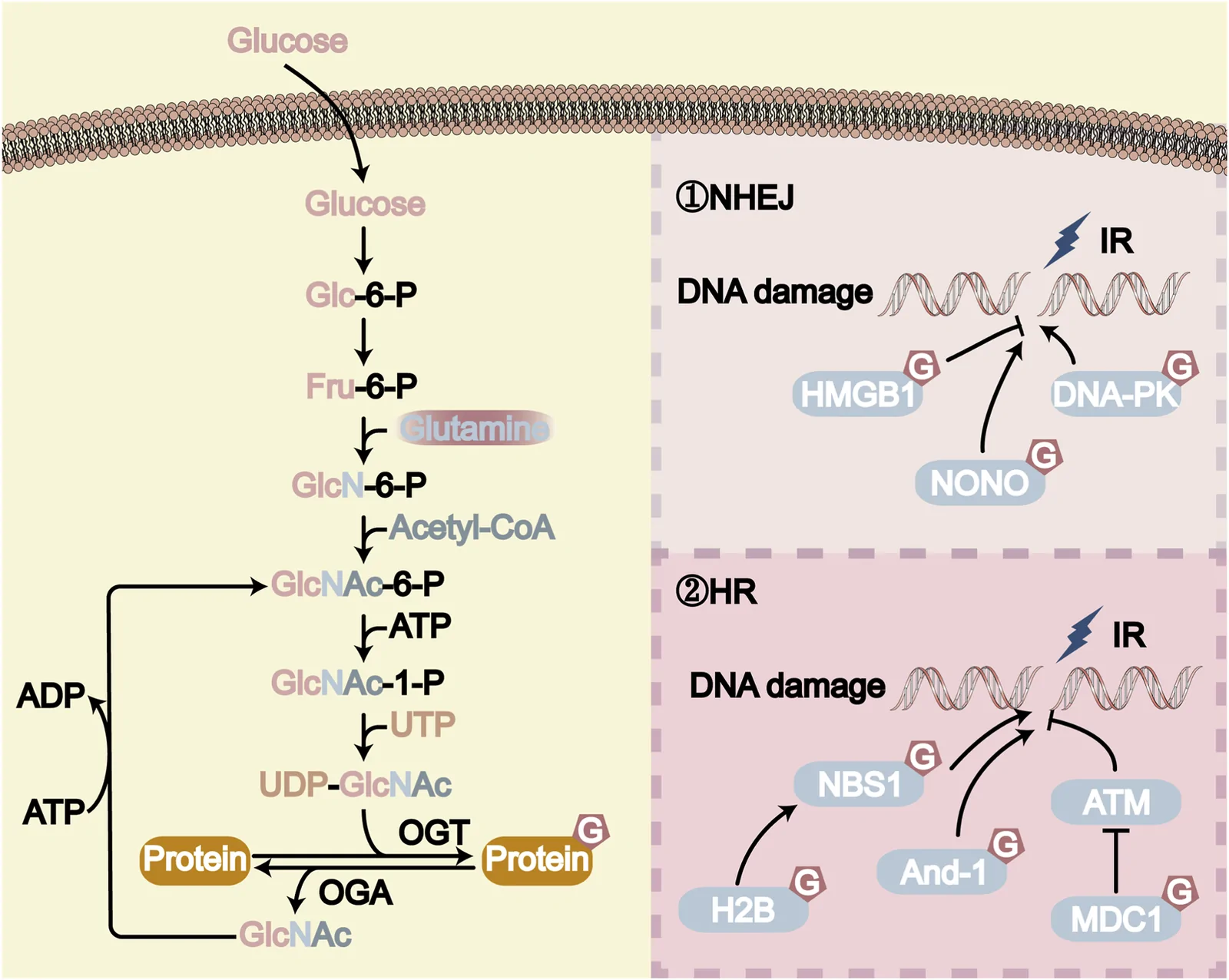
Molecular mechanisms by which glutamine metabolism regulates O-GlcNAcylation of DNA damage repair proteins. Glutamine mediates UDP-GlcNAc production via the hexosamine biosynthetic pathway (HBP), establishing a dynamic balance of O-GlcNAcylation in tumor cells through the opposing actions of O-linked N-acetylglucosaminyltransferase (OGT) and O-linked N-acetyl β-D-glucosaminidase (OGA). O-GlcNAcylation modulates the activity of key DNA repair proteins in a substrate-specific manner: in the nonhomologous end-joining (NHEJ) pathway, O-GlcNAcylation of DNA-PK and NONO promotes repair, while O-GlcNAcylation of high-mobility group box 1 (HMGB1) inhibits its pro-repair function; in the homologous recombination (HR) pathway, O-GlcNAcylation of Nijmegen breakage syndrome protein 1 (NBS1) and acidic nucleoplasmic DNA-binding protein 1 (And-1) enhances DSB repair, whereas O-GlcNAcylation of mediator of DNA damage checkpoint 1 (MDC1) competitively inhibits its phosphorylation and impairs ATM kinase recruitment to damage sites. Core histones (H2A, H2B, H3, H4) can also undergo O-GlcNAcylation, with H2B serine 112 modification shown to promote NBS1 recruitment to DSB sites. UDP-GlcNAc, uridine diphosphate N-acetylglucosamine; GFAT, glutamine-fructose-6-phosphate amidotransferase; Fru-6-P, fructose-6-phosphate; GlcN-6-P, glucosamine-6-phosphate; GlcNAc-6-P, N-acetylglucosamine-6-phosphate; GlcNAc-1-P, N-acetylglucosamine-1-phosphate; OGT, O-linked N-acetylglucosaminyltransferase; OGA, O-linked N-acetyl β-D-glucosaminidase; HR, homologous recombination; NHEJ, non-homologous end joining; MDC1, mediator of DNA damage checkpoint 1; ATM, ataxia telangiectasia mutated; DNA-PK, DNA-dependent protein kinase; NONO, non-POU domain-containing octamer-binding protein; HMGB1, high-mobility group box 1; And-1, acidic nucleoplasmic DNA-binding protein 1; NBS1, Nijmegen breakage syndrome protein 1.

#### 4.2.2 O-GlcNAcylation exerts significant functional impacts on DNA repair proteins

During DDR, OGT is rapidly recruited to DNA damage sites induced by various genotoxic agents, including doxorubicin and ultraviolet rays, though evidence specifically for IR-induced damage remains limited, and participates in the regulation of DNA damage repair by locally catalyzing O-GlcNAcylation (Na et al., 2020; Chen and Yu, 2016). In breast cancer cells and human cervical epithelial cancer cell lines, inhibition of OGT expression significantly enhanced cellular radiosensitivity, whereas inhibition of OGA expression increased cellular O-GlcNAcylation levels and radiation resistance, as verified by colony formation experiments (Efimova et al., 2019; Averbek et al., 2021). These findings, together with the established dependence of O-GlcNAcylation on UDP-GlcNAc availability from the HBP, raise the possibility that glutamine may ultimately regulate cellular radiosensitivity and DNA damage repair capacity by modulating the HBP pathway-mediated UDP-GlcNAc synthesis and O-GlcNAcylation. This hypothesis provides a conceptual framework for understanding the cross-regulation of metabolic reprogramming and the DNA damage response. After treatment with IR in tumors, the levels of O-GlcNAcylation of several key proteins involved in DNA damage repair were significantly altered, such as And-1, CtIP, and RAD51, which in turn dynamically regulated the activation and progression of the DNA damage repair network (Averbek et al., 2021; Zhou et al., 2022). HR and NHEJ are two central mechanisms for cellular repair of IR-induced DSBs. The following section will detail the functional alterations and regulatory mechanisms of several core repair proteins upon O-GlcNAcylation.

The DNA-dependent protein kinases (DNA-PK) complex (including DNA-PKcs and Ku70/80 heterodimer) plays a central role in NHEJ. When DSBs occur, Ku70/80 rapidly recognizes and binds to the broken ends, and subsequently recruits repair factors such as DNA-PKcs to initiate the NHEJ repair process. NHEJ repair activity is regulated by the O-GlcNAcylation of DNA-PK (Lafont et al., 2020) and Ku70/80 (Cui et al., 2021). A study using a HeLa cell model showed that the level of O-GlcNAcylation of DNA-PKcs was closely related to its kinase activity; lowering O-GlcNAcylation significantly inhibited DNA-PKcs activity, whereas increasing this modification did not affect this function (Lafont et al., 2020). Nuclear high-mobility group box 1 (HMGB1) is a non-histone chromatin-associated structural protein that has a key role in maintaining DNA repair and chromosomal stability in the nucleus (Tang et al., 2023; Balana et al., 2021). On the one hand, HMGB1 has been implicated in recruiting DNA-PK and promoting NHEJ, although the precise mechanism remains incompletely understood (Lange and Vasquez, 2009). Notably, O-GlcNAcylation of HMGB1 has been reported to attenuate its association with repair complexes, leading to DNA damage accumulation (Balana et al., 2021). Collectively, the regulation of DNA damage repair by HMGB1 via the NHEJ pathway may be related to O-GlcNAcylation levels. This potential association requires further in-depth investigation and experimental verification. In addition, following OGT-mediated O-GlcNAcylation of NONO, timely removal of this modification by OGA is required for NONO to effectively recruit downstream repair factors, which in turn promotes the cascade reaction of DNA damage repair. Additionally, studies have shown that inhibition of deglycosylation of the NHEJ-associated protein NONO prolongs its retention time at DNA damage sites, which may delay its degradation and impair NHEJ repair (Cui et al., 2021).

Nijmegen breakage syndrome protein 1 (NBS1), a regulatory protein of the HR pathway, has been shown to undergo O-GlcNAcylation. Although this does not affect its initial recruitment process to DSBs, it significantly prolongs the retention time of NBS1 on DSBs and enhances its binding stability. This indicates that the inhibition of NBS1 O-GlcNAcylation significantly hinders the HR repair pathway (Averbek et al., 2021). Another regulatory protein of the HR pathway, acidic nucleoplasmic DNA-binding protein 1 (And-1), promotes DSB repair through the following mechanisms: 1) recruitment to DSB sites, 2) promotion of CtIP recruitment, and 3) enhancement of the CtIP-NBS1 interaction. Studies have shown that the O-GlcNAcylation significantly promoted the accumulation of And-1 to DSB sites, suggesting that this modification may enhance the efficiency of HR-mediated DSB repair and escalate the radiation resistance of tumor cells (Zhou et al., 2022). Studies have shown that O-GlcNAcylation and phosphorylation can compete for the same serine/threonine residues in a site-specific manner. Elevated O-GlcNAcylation of MDC1 inhibits its phosphorylation through the mechanism of site competition, which in turn hinders the recruitment of ATM, ultimately leading to impairment of DNA damage repair (Chen and Yu, 2016). In summary, O-GlcNAcylation/deglycosylation dynamically regulates the activity of key DDR factors, thereby fine-tuning the balance and efficiency of the HR and NHEJ repair pathways.

The spatial conformation of chromatin directly affects the functions of DNA damage repair proteins. As the basic structural units of chromatin, core histones (H2A, H2B, H3, and H4) dynamically regulate chromatin structure through various PTMs (Gao et al., 2025; Ai et al., 2022). Dynamic chromosomal movement is necessary to initiate effective DNA repair, whereas DNA damage itself can induce conformational changes in chromosomes to form a microenvironment favorable for repair (Tian et al., 2025). Notably, all core histones can undergo O-GlcNAcylation (Xu et al., 2022a; Wang et al., 2015). Although previous research revealed that the O-GlcNAcylation of NBS1 itself does not affect its recruitment to DSB sites, other studies have shown that O-GlcNAcylation of histone H2B serine 112 (S112) significantly promotes this process (Wang et al., 2015). This finding suggests that the targeted modulation of O-GlcNAcylation may be a potential therapeutic strategy to enhance tumor radiosensitivity.

O-GlcNAcylation modulates DNA damage repair in a substrate-specific manner, with some modifications promoting and others inhibiting repair enzyme recruitment to damage sites (Efimova et al., 2019; Wang et al., 2015; Hayakawa et al., 2017). These studies have revealed the dual regulatory role of O-GlcNAcylation in the DNA damage response; both roles affect the stability and activity of repair enzymes and regulate the repair microenvironment by altering chromatin structure (Table 1). These findings highlight the substrate-specific directionality of O-GlcNAcylation effects. While reducing O-GlcNAcylation of repair-promoting substrates such as DNA-PKcs and And-1 may impair their repair functions and sensitize tumor cells to IR, enhancing O-GlcNAcylation of substrates whose modification inhibits repair, such as HMGB1 or MDC1, could achieve the same radiosensitizing effect through an opposite mechanism. This heterogeneity underscores the need for substrate-selective modulation rather than global OGT or OGA inhibition. It is noteworthy that many of the studies establishing O-GlcNAcylation-DDR connections employed genotoxic agents such as doxorubicin or ultraviolet radiation rather than ionizing radiation. Although shared downstream signaling provides a basis for extrapolation, IR-induced clustered DNA damage and water radiolysis-derived ROS may engage O-GlcNAcylation-dependent repair regulation through distinct mechanisms.

**TABLE 1:** Dual regulatory mechanisms of O-GlcNAcylation in DDR: from enzyme modification to chromatin remodeling.

Classification	Protein	Trigger	Effect	References
NHEJ	DNA-PKcs	camptothecin or bleomycin treatment	O-GlcNAcylation of DNA-PKcs was positively correlated with its kinase activity	lafont et al. (2020)
	NONO	IR	Inhibiting the deglycosylation of O-GlcNAcylated NONO impairs NHEJ.	Cui et al. (2021)
HR	NBS1	IR	O-GlcNAcylation of NBS1 enhances its binding stability to DSBs to ensure the HR repair pathway	Averbek et al. (2021)
	And-1	IR	O-GlcNAcylation promotes And-1 accumulation at DNA break sites to recruit downstream repair proteins, thereby enhancing HR repair and radiation resistance	Zhou et al. (2022)
	MDC1	IR, etoposide, cisplatin among others	O-GlcNAcylation of MDC1 inhibits its phosphorylation modification, which in turn hinders ATM kinase recruitment	Chen and Yu (2016)
Histone	H2B	IR, methyl methanesulfonate among others	H2B undergoes O-GlcNAcylation followed by significant promotion of NBS1 recruitment toward DNA breakpoints	Wang et al. (2015)

### 4.3 Glutamine regulates the PARylation of proteins associated with DNA damage repair

Glutamine metabolism indirectly modulates cellular nicotinamide adenine dinucleotide (NAD^+^) homeostasis (Du et al., 2025; Liu P-S. et al., 2023), which serves as an essential substrate for poly (ADP-ribose) polymerases (PARPs), which catalyze ADP-ribosylation to mediate DNA damage repair (Svetlova et al., 2023). NAD^+^ is synthesized from tryptophan via the kynurenine/quinolinic acid pathway, regenerated from nicotinamide through the salvage pathway or Preiss Handler Pathway (PH) from nicotinic acid (Xu et al., 2022b; Chowdhry et al., 2019). Although glutamine is not a canonical NAD^+^ precursor, glutamine-derived metabolites support NAD^+^ biosynthesis and recycling through multiple interconnected mechanisms. First, glutamine metabolism indirectly supports the NAD^+^ salvage pathway by sustaining PPP flux and thus the availability of PRPP, which serves as an essential co-substrate for the nicotinamide phosphoribosyltransferase (NAMPT)-catalyzed rate-limiting step that condenses nicotinamide with PRPP to generate nicotinamide mononucleotide (NMN) (Ratia et al., 2023; Pol et al., 2021). Second, by fueling TCA cycle anaplerosis and subsequent oxidative phosphorylation, glutamine metabolism provides the ATP required for NAD^+^ biosynthesis (Kami Reddy et al., 2024; Zei et al., 2022). Third, PARP1 activation following DNA damage can consume a large amount of nuclear NAD^+^ pools, necessitating rapid regeneration supported by glutamine-derived ATP (Wang et al., 2023; Sarkar et al., 2022). Fourth, beyond metabolite supply, glutamine fuels O-GlcNAcylation of phosphoribosyl pyrophosphate synthetase 1 (PRPS1) via the hexosamine biosynthetic pathway, thereby enhancing PRPS1 enzymatic activity and PRPP output, which in turn amplifies NAD^+^ production through the salvage pathway (Chen et al., 2023c; Hopp et al., 2021; Güldenpfen et al., 2023; Rudolph et al., 2021; Gibson and Kraus, 2012). Collectively, glutamine sustains NAD^+^ availability and PARylation capacity not as a direct biosynthetic precursor, but through metabolic flux, PRPP supply, and post-translational regulation of key salvage enzymes. Systematic elucidation of this glutamine-NAD^+^-PARylation axis may provide important insights into understanding the interplay between cellular metabolism and genomic stability.

#### 4.3.1 Poly (ADP-ribose) polymerases (PARPs)

The PARP family comprises 17 distinct proteins encoded by different genes (Lei et al., 2020), playing a crucial role in DNA damage recognition and DDR (Blessing et al., 2022; Robu et al., 2025). Among them, PARP1 and PARP2 are strongly activated by SSBs (Sefer et al., 2022). They catalyze the transfer of ADP-ribose polymers to substrates, thereby participating in the regulation of DNA damage-induced cellular responses and chromatin structure (Wu et al., 2021; Galindo-Campos et al., 2022). Moreover, these three PARP proteins can recruit DNA repair proteins to damage sites via pathways such as HR and NHEJ, initiating the repair of SSB, BER, NER, and DSB (Huang and Kraus, 2022). As the most extensively studied member of the PARP family, PARP1 modulates repair processes through PARylation of various DNA repair-related proteins, including XRCC1 (scaffold protein for other DNA repair enzymes) (Hrychova et al., 2024), histones (Brustel et al., 2022), and non-histone chromatin-associated proteins (Bacic et al., 2024).

In the context of radiotherapy, PARP1 serves as a critical DNA damage sensor that coordinates the repair of the abundant IR-induced SSBs via base excision repair, while simultaneously acting as a cell cycle-dependent molecular switch at DSB sites: by competing with the Ku70/Ku80 complex for DNA end access and orchestrating PARylation-dependent recruitment of repair mediators such as BRCA1 and the MRN complex, PARP1 dynamically channels DSB repair toward either error-free HR in S/G2 phase or error-prone NHEJ predominantly in G1 phase (Zhao X. et al., 2023; Efimova et al., 2026; Pan et al., 2023). Notably, the NAD^+^ supply required for sustained PARP1 activity following IR is metabolically supported by glutamine-driven NAD^+^ biosynthesis (as discussed above), establishing a functional link between glutamine metabolism and PARylation-dependent DNA damage repair (Tyagi and Das, 2024; Panizza et al., 2023; Xiang et al., 2024).

#### 4.3.2 PARylation modulates the function of histones and non-histone proteins

Histone PARylation directly participates in the DNA repair process by modulating chromatin structure and function (Brustel et al., 2022; Hananya et al., 2021). Specifically, PARylation of histone H1 induces relaxation of the chromatin superstructure, thereby facilitating the recruitment of DNA repair enzymes to damage sites (Bonfiglio et al., 2017; Palazzo et al., 2018; Koshkina et al., 2025). Meanwhile, PARylation of histone H2B, via its ADP-ribose interaction domain, promotes chromatin remodeling and enriches relevant repair factors in response to DSBs (Bacic et al., 2024; Gibbs-Seymour et al., 2016). These mechanisms demonstrate that histone PARylation not only plays a vital physiological role in chromatin assembly but also provides crucial chromatin accessibility for DNA repair proteins.

In addition to histones, chromatin binds to a variety of non-histone proteins. These proteins are typically acidic and play crucial roles not only in the dynamic changes of chromatin structure but also in regulating the spatial conformation and functional state of DNA (Bastié et al., 2024; Li F. et al., 2025). Among them, chromatin remodeling factors such as amplified in liver cancer 1 (ALC1, also known as chromodomain-helicase-DNA-binding protein 1-like, CHD1L) are key regulatory proteins in the DNA damage response. ALC1 can be activated by poly (ADP-ribose) deposition induced by DNA damage and specifically recognize PARylation signals at damage sites, thereby initiating local chromatin remodeling (Bacic et al., 2024; Bacic et al., 2021). At DNA damage sites, the PARylation of ALC1 induces chromatin relaxation and promotes the recruitment of other chromatin remodeling factors, such as chromodomain helicase DNA binding protein 3 (CHD3) and chromodomain helicase DNA-binding protein 4 (CHD4), collectively facilitating the repair process (Smith et al., 2018). Notably, the activation of ALC1 may depend on histone PARylation mediated by histone PARylation factor 1 (HPF1) (Bacic et al., 2024; Bacic et al., 2021). Additionally, histone PARylation plays a critical role in guiding the directional recruitment of ALC1 to nucleosomes adjacent to damage sites and regulating its sliding direction (Bacic et al., 2024). Beyond the ALC1 pathway, PARP1 and its mediated PARylation also regulate chromatin architecture by influencing the multifunctional chromatin architecture protein CCCTC-binding factor (CTCF) (Guastafierro et al., 2013). Although the recruitment of CTCF to DNA damage sites is independent of PARylation, CTCF itself can be highly PARylated, acting as a scaffold protein to recruit and stabilize repair factors such as BRCA2, thereby promoting efficient damage repair (Hilmi et al., 2017) (Figure 5).

**FIGURE 5:**
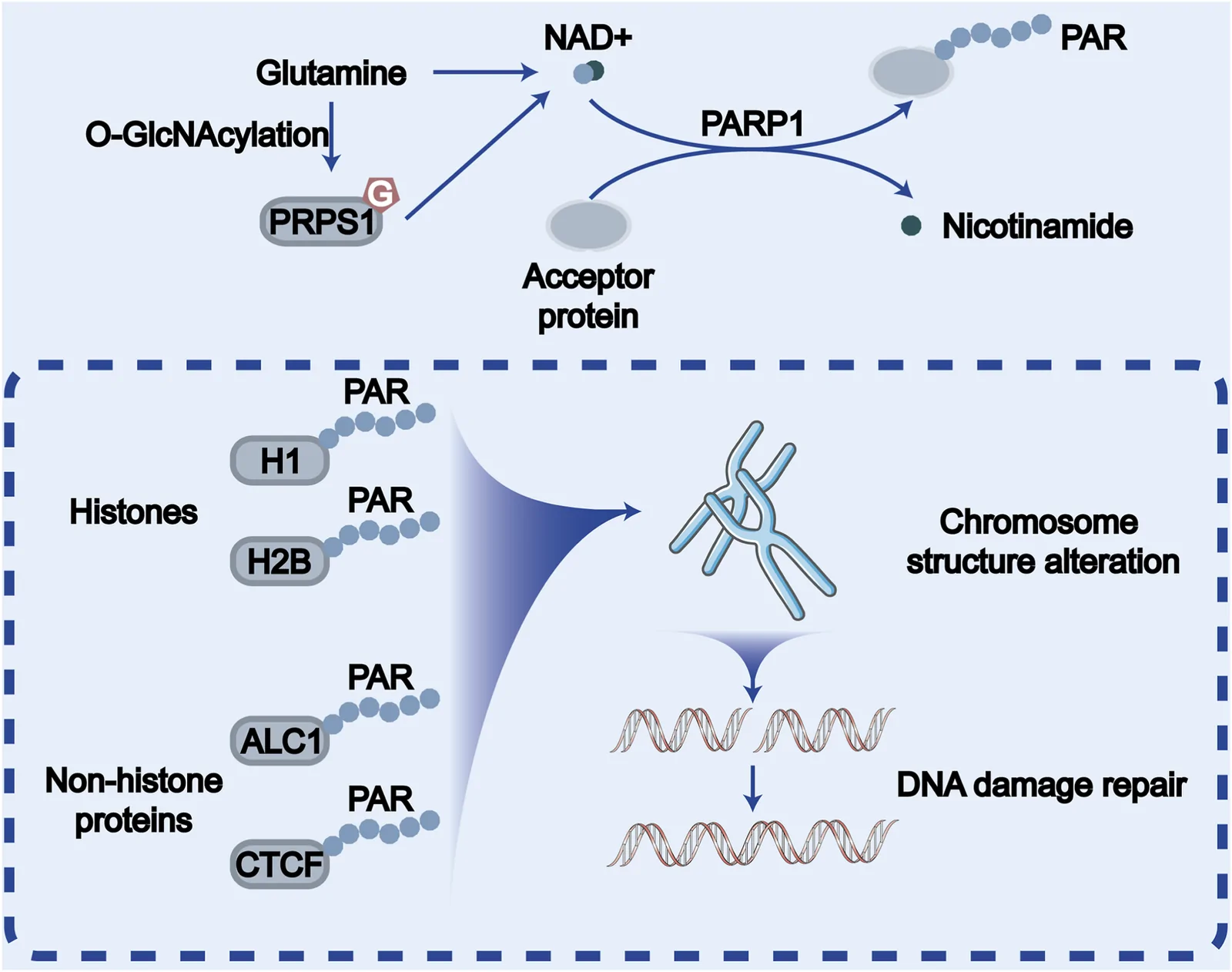
Molecular mechanisms and DNA damage repair by which glutamine metabolism regulates PARylation. Glutamine regulates NAD^+^ production through a dual mechanism: on one hand, it serves as a metabolic substrate directly involved in NAD^+^ synthesis; on the other hand, it modulates the O-GlcNAcylation of PRPS1 to influence its enzymatic activity, thereby indirectly contributing to NAD^+^ generation. In the context of chromatin structure remodeling, histones H1 and H2B, as well as non-histone proteins ALC1 and CTCF, undergo PARylation, a modification that facilitates chromatin relaxation and provides the necessary spatial basis for subsequent DNA damage repair. NAD^+^: nicotinamide adenine dinucleotide; PRPS1, phosphoribosyl pyrophosphate synthetase 1; PAR, poly (ADP-ribose); PARP-1, poly (ADP-ribose) polymerase 1; ALC1, amplified in liver cancer 1; CTCF, CCCTC-binding factor.

The PARP family participates in regulating multiple DNA damage repair pathways by directly recognizing DNA damage sites and catalyzing PARylation of itself and other proteins. Glutamine, by sustaining NAD^+^ availability through metabolic flux rather than serving as a direct biosynthetic precursor, may modulate DNA damage repair by influencing the PARylation, thereby affecting tumor response to radiotherapy. Therefore, further investigation into how glutamine influences DNA damage repair through PARylation may offer novel targets for enhancing tumor radiosensitivity.

### 4.4 Glutamine metabolism modulates oxidative stress and redox homeostasis in irradiated tumor cells

Ionizing radiation kills tumor cells largely through the generation of ROS, which cause DNA damage, lipid peroxidation, and protein oxidation (Yang P. et al., 2023; Hu et al., 2020). The ability of tumor cells to counteract IR-induced oxidative stress is therefore a critical determinant of radiosensitivity (Yue et al., 2025; Luo et al., 2025). Glutamine metabolism supports cellular antioxidant defense through multiple interconnected mechanisms, such as GSH synthesis, NADPH regeneration, and cystine import via solute carrier family 7 member 11 (SLC7A11, also called xCT) (Zhu et al., 2022; Ying et al., 2021; Liu et al., 2020).

#### 4.4.1 Glutamine supports GSH synthesis and NADPH production to counteract IR-induced ROS

Intracellular glutamine is converted to glutamate by GLS (Jiang et al., 2025). Glutamate then serves two complementary antioxidant functions (Choi et al., 2025; Badgley et al., 2020). First, glutamate is one of the three amino acid precursors (glutamate, cysteine, and glycine) required for GSH synthesis, catalyzed sequentially by glutamate‐cysteine ligase catalytic subunit (GCLC) and GS (Bai et al., 2025; Kalinina, 2024). GSH is one of the most abundant intracellular thiols and a primary scavenger of IR-induced ROS (Liu R. et al., 2022). Second, intracellular glutamate serves as the substrate for system xc^−^ (also known as the cystine/glutamate antiporter), which is composed of the light chain subunit SLC7A11 (xCT) and the heavy chain subunit SLC3A2 (Koppula et al., 2020). System xc^−^ exchanges intracellular glutamate for extracellular cystine (the oxidized disulfide form of cysteine) (Parker et al., 2021). Imported cystine is subsequently reduced to cysteine by intracellular reductants and utilized for GSH synthesis (Martí-Andrés et al., 2024). Thus, glutamine-derived glutamate indirectly supports the availability of cysteine, the rate-limiting amino acid for GSH synthesis, by driving cystine import through system xc^−^ (Zhang et al., 2022). Experimental evidence has demonstrated that glutamate availability, xCT-mediated cystine uptake, and GSH/redox homeostasis are functionally interconnected, and disruption of this axis sensitizes tumor cells to oxidative stress (Zhou et al., 2024; Liu X. et al., 2023; Yin et al., 2024).

#### 4.4.2 Redox regulation by glutamine metabolism influences DNA damage persistence and repair efficiency after IR

GSH synthesis alone does not fully account for cellular antioxidant capacity (Aurora et al., 2022; Zhang et al., 2023; Mishima et al., 2022). Following ROS scavenging, glutathione disulfide (GSSG) must be regenerated to reduced GSH by glutathione reductase (GR), a reaction that strictly requires NADPH as a cofactor (Brenner et al., 2026). Therefore, NADPH availability is equally critical for maintaining the GSH/GSSG ratio and overall redox buffering capacity (Lan et al., 2024).

Glutamine carbon contributes to NADPH generation through multiple metabolic routes. Through glutamate dehydrogenase 1 (GDH1), glutamine-derived glutamate undergoes oxidative deamination to α-KG, and importantly, this reaction can utilize NADP^+^ as an electron acceptor to generate NADPH (Jin et al., 2015; Mi et al., 2025), thereby linking glutaminolysis to cellular redox homeostasis and antioxidant defense. In KRAS-mutant tumors such as pancreatic cancer, the aspartate transaminase (GOT1)/malate dehydrogenase (MDH1)/malic enzyme 1 (ME1) axis constitutes a major pathway for glutamine-derived NADPH production. In this route, glutamate is transaminated to aspartate, which is subsequently converted to oxaloacetate and then reduced to malate, and ME1 finally catalyzes the NADP^+^-dependent oxidative decarboxylation of malate to pyruvate, releasing CO_2_ and generating NADPH in the process (Radyk et al., 2026; Son et al., 2013). Additionally, the pentose phosphate pathway (PPP) is also the main source of NADPH for cancer cells to combat oxidative stress (Ding et al., 2021). Although it is mainly driven by glucose, it works in a mutually compensatory manner with the aforementioned NADPH production pathway derived from glutamine (Ying et al., 2021). Impairment of PPP flux upregulates glutaminolysis and ME1-dependent NADPH generation (Aurora et al., 2022), while loss of ME1 redirects glucose carbon into the PPP (Brashears et al., 2022), collectively ensuring a robust supply of cellular reducing equivalents.

These pathways collectively ensure that glutamine metabolism supports not only GSH synthesis but also the NADPH-dependent regeneration of reduced GSH, thereby maintaining redox homeostasis following IR-induced oxidative stress.

#### 4.4.3 Context-dependent relationships with ferroptosis

Disruption of the glutamine/glutamate/cystine/GSH axis, whose contributions to GSH biosynthesis and NADPH-dependent redox recycling are detailed in Sections 4.4.1, 4.4.2, impairs lipid peroxide detoxification and sensitizes cells to ferroptosis (Allevato et al., 2024; Shen et al., 2025), a form of regulated cell death driven by iron-dependent lipid peroxidation (Dixon et al., 2012). The terminal checkpoint of ferroptotic execution is governed by glutathione peroxidase 4 (GPX4), which utilizes GSH as a reducing substrate to reduce membrane phospholipid hydroperoxides (PL-OOHs) to corresponding alcohols, thereby aborting the iron-catalyzed radical chain propagation that would otherwise culminate in plasma membrane rupture (Hu et al., 2025; Ramos et al., 2024; Sassano et al., 2025).

Through its conversion to glutamate, glutamine simultaneously fuels GSH biosynthesis, thereby supplying the GSH required as the reducing substrate for GPX4 activity, and supplies α-KG to maintain TCA cycle flux (Bai et al., 2025; Shi et al., 2024). Glutamine thus occupies a dual position within the ferroptosis circuit. Under cystine-replete conditions, glutamine-derived GSH is predominantly cytoprotective, preserving GPX4 function and preventing the accumulation of lethal PL-OOH species (Ge et al., 2025). However, under cystine-deprived conditions, excessive glutaminolysis can drive TCA cycle hyperactivation and mitochondrial ROS production while simultaneously depleting GSH reserves, thereby tipping the balance from antioxidant defense toward unchecked lipid peroxidation and ferroptosis (Li et al., 2021; Feng et al., 2022).

These considerations carry direct implications for radiation oncology. IR generates secondary ROS bursts through mitochondrial dysfunction and metabolic reprogramming that can trigger ferroptosis in metabolically susceptible cells (Yang P. et al., 2023; Qian et al., 2026). Glutamine-targeting strategies under investigation as radiosensitizers must therefore carefully consider the nutrient landscape of the tumor microenvironment, particularly the local availability of cystine and glutamine, given that the therapeutic impact on ferroptosis susceptibility may diverge substantially across distinct metabolic contexts.

## 5 Targeting the glutamine metabolic pathway as a therapeutic strategy to enhance genotoxic treatment efficacy

As a key node molecule in tumor metabolic reprogramming, glutamine plays an important role in the proliferation, survival, invasion, and metastasis of malignant tumors. Several studies have confirmed that targeting key enzymes or transporters in the glutamine metabolic pathway, such as glutaminase (Tran et al., 2017) and ASCT2 (Hassan et al., 2013), has been shown to inhibit tumor cell growth in preclinical models, suggesting potential as an interventional strategy for cancer therapy. The following sections will systematically elaborate on the role of targeting glutamine metabolism in tumor treatment.

### 5.1 Targeting ASCT2 has emerged as a crucial therapeutic strategy

Two inhibitors targeting the glutamine transporter protein alanine-serine-cysteine transporter 2 (ASCT2), the competitive inhibitor γ-L-glutamyl-p-nitroanilide (GPNA) and the selective inhibitor V-9302, both significantly inhibited cell proliferation and promoted cell death by elevating intracellular ROS levels in various tumor models, including those of lung and breast cancer (Hassan et al., 2013; Schulte et al., 2018). Notably, the combination of V-9302 with photosensitizers chlorin e6 (Ce6) and pyropheophorbide ‘a’ (PPa) synergistically enhanced ROS production and significantly improved antitumor efficacy (Zhao L. et al., 2023; Jiang Q. et al., 2023).

In summary, current studies indicate that the main focus of intervention should be to inhibit glutamine transport using ASCT2 inhibitors such as GPNA and V-9302 (Hassan et al., 2013; Schulte et al., 2018), which significantly inhibit tumor cell growth. Interestingly, some inhibitors have demonstrated favorable synergistic effects in preclinical studies, particularly noted with DNA-damaging agents such as methyl methanesulfonate (MMS) or photodynamic therapy (PDT) (Tran et al., 2017; Wang Q. et al., 2024). This combination therapy effectively enhanced the antitumor effects of monotherapies. While these synergistic effects were observed with PDT rather than ionizing radiation, the shared mechanism of ROS-mediated cytotoxicity provides a rationale for exploring ASCT2 inhibition in combination with radiotherapy.

### 5.2 Targeting glutamine metabolic enzymes has emerged as a promising strategy in cancer therapy

In neuroblastoma and breast cancer cells, amino oxyacetate (AOA), an inhibitor of glutamate-dependent transaminase, and epigallocatechin gallate (EGCG), an inhibitor of glutamate dehydrogenase (GDH), dose-dependently induce apoptosis, with potency comparable to that of glutamine deprivation (Qing et al., 2012; Thornburg et al., 2008). Diazo-5-oxo-L-norleucine (DON) is a glutamine analog that functions as a broad-spectrum glutamine antagonist, rather than a selective glutaminase inhibitor (Encarnación-Rosado et al., 2023), which triggered DNA damage in a prostate cancer model with treatment effects similar to those of glutamine starvation. Notably, *in vitro* experiments have shown that DON combined with MMS synergistically enhanced DNA damage levels, and *in vivo* experiments further confirmed that this combination therapy was more effective against tumors than monotherapy (Tran et al., 2017). Although these studies employed the alkylating agent MMS rather than ionizing radiation, the shared endpoint of DNA damage accumulation provides a mechanistic rationale for testing DON in combination with radiotherapy. In addition, although the glutaminase inhibitor HYL001 has limited antitumor effects alone, its combination with the photosensitizer CyOA (hemicyanine-oleic acid conjugate) has significantly enhanced tumor cell lysis by synergistically elevating ROS levels (Wang Q. et al., 2024).

### 5.3 Combining glutamine metabolism inhibitors with radiotherapy enhances therapeutic efficacy

While glutamine metabolism inhibitors have shown promising results when combined with chemotherapy and photodynamic therapy (as described in Sections 5.1, 5.2), their efficacy as radiosensitizers requires careful evaluation. Unlike PDT, which generates ROS through photosensitizer-light interactions, or alkylating agents like MMS, which directly damage DNA bases, ionizing radiation induces a broader spectrum of DNA lesions and elicits a more complex DNA damage response. The metabolic vulnerabilities exploited by glutamine-targeting agents may therefore have distinct implications for radiosensitization compared to other genotoxic modalities. Building on the preclinical evidence from chemotherapy and photodynamic combinations described above, recent studies have specifically investigated glutamine metabolism inhibitors as radiosensitizers. Among these, CB-839 is the most clinically advanced GLS1 inhibitor and has demonstrated radiosensitizing effects in models of prostate cancer, head and neck squamous cell carcinoma, and soft-tissue sarcoma (Mukha et al., 2021a; Mukha et al., 2021b; Patel et al., 2024; Wicker et al., 2021). Another GLS1 inhibitor, BPTES, has also been shown to regulate the radiosensitivity of tumor cells in hepatocellular carcinoma models (Wang et al., 2025b). Furthermore, although clinical trials of the GLS inhibitors, NCT02861300 and NCT02828217, have preliminarily proven safe, their exact therapeutic efficacy requires further validation (Jiang et al., 2025). Beyond targeting GLS, the inhibitor V-9302, which acts on the transporter protein ASCT2, has exhibited similar radiosensitizing effects in mouse models of head and neck squamous cell carcinoma (Song et al., 2024).

Given the critical role of glutamine metabolism in regulating DNA damage repair, targeted inhibition of this pathway has emerged as a highly promising preclinical sensitization strategy. By disrupting the metabolic reprogramming of tumor cells and impairing their DNA repair capacity, this approach aims to enhance the antitumor efficacy of existing therapies such as radiotherapy, chemotherapy, and targeted therapy, thereby opening new avenues for combination treatment (Table 2). Future research should focus on the following areas: First, the context-dependent nature of ferroptosis regulation by glutamine metabolism (Section 4.4.3) necessitates biomarker-driven patient selection, particularly assessment of tumor microenvironment cystine and glutamine availability, before clinical deployment of glutamine-targeting radiosensitizers. Second, the compensatory role of nucleotide salvage pathways (Section 4.1.3) suggests that dual targeting of *de novo* synthesis and salvage may be required to achieve meaningful radiosensitization. Third, the substrate-specific directionality of O-GlcNAcylation effects on DDR proteins (Section 4.2.2) argues for substrate-selective modulation rather than global OGT/OGA inhibition to avoid unintended consequences on normal tissue repair. Fourth, the glutamine-NAD^+^-PARylation axis (Section 4.3) reveals a potentially exploitable vulnerability: since glutamine metabolism sustains the NAD^+^ pool required for PARP catalytic activity, combining glutamine pathway inhibitors with PARP inhibitors may achieve dual suppression of both NAD^+^ supply and PARP-mediated repair, which is a metabolic-pharmacological synthetic lethal strategy that warrants systematic investigation.

**TABLE 2:** Therapeutic targeting of glutamine metabolism to enhance genotoxic treatment efficacy.

Classification	Tumor type	Drug	Effect of inhibition of glutamine metabolism	Combination therapy with genotoxic or radiation treatment	References
ASCT2 inhibitors	lung cancer cell	γ-L-glutamyl-p-nitroanilide (GPNA)	The pharmacological targeting of ASCT2 decreased cell growth and viability in lung cancer cells	-	Hassan et al. (2013)
	mammary squamous cell	V-9302	V-9302 blocks ASCT2 function, resulting in attenuated cancer cell growth and proliferation, increased oxidative stress and cell death, which contributes to tumor therapy	V-9302 significantly enhanced the photodynamic antitumor efficacy mediated by Ce6 and PPa by inducing intracellular ROS accumulation	Schulte et al. (2018), Zhao et al. (2023b), Jiang et al. (2023b)
Inhibitors of glutamate-dependent transaminases	lung cancer cell and breast cancer cell	Aminooxyacetate (AOA)	AOA treatment inhibits tumor growth	-	Qing et al. (2012), Thornburg et al. (2008)
Inhibitors of glutamate dehydrogenase (GDH)	neuroblastoma cell	Epigallocatechin gallate (EGCG)	EGCG, which effectively induced apoptosis at comparable levels to that resulting from Gln starvation, induces cell death in a dose-dependent manner in human neuroblastoma cell lines	-	Qing et al. (2012)
Glutaminase inhibitor	prostate cancer, head and neck squamous cell carcinoma, and soft-tissue sarcoma	CB-839	CB-839 inhibits GLS1 and depletes intracellular glutamine	Radiosensitizing effects have been demonstrated in preclinical models	Mukha et al. (2021a), Mukha et al. (2021b), Patel et al. (2024), Wicker et al. (2021)
	prostate cancer	6-Diazo-5-oxo-L-norleucine (DON)	DON treatment triggers DNA damage effects similar to glutamine starvation	DON in combination with MMS synergistically enhances DNA damage levels, significantly inhibits tumor growth and reduces tumor weight	Tran et al. (2017)
	breast cancer stem cells (BCSCs)	HYL001	HYL001 increases intracellular ROS levels	HYL001, when combined with the photosensitizer CyOA, significantly enhances tumor cell killing by synergistically increasing ROS levels	Wang et al. (2024b)
	hepatocellular carcinoma	BPTES	BPTES inhibits the activity of glutamine synthetase GLS1, blocking the conversion of glutamine to glutamate	BPTES enhanced tumor response to radiation	Wang et al. (2025b)

## 6 Conclusion

As radiotherapy plays an increasingly prominent role in cancer treatment, tumor radioresistance has become a key bottleneck limiting its efficacy. Glutamine is a multifunctional metabolic substrate that serves as an energy source for cellular biological activities and provides carbon and nitrogen for the synthesis of many biomolecules. More importantly, glutamine is deeply involved in tumor proliferation and DNA damage repair by providing energy and DNA repair substrates as well as regulating the network of protein PTMs, such as O-GlcNAcylation and PARylation. Therefore, targeting glutamine metabolism to enhance radiosensitivity has emerged as a closely watched strategy in translational research. Studies have shown that glutamine deprivation or inhibition of glutamine metabolism can lead to a significant decrease in the cellular stress response capacity, manifested by oxidative damage accumulation and DNA repair dysfunction, which in turn significantly enhance the radiosensitivity of tumor cells. Although some glutamine metabolism inhibitors have demonstrated acceptable safety profiles, their clinical efficacy remains inadequately supported by evidence, highlighting the urgent need to advance related clinical studies. In conclusion, the development of highly selective glutamine metabolism inhibitors, or their combination with existing radiotherapy regimens, holds promise for offering novel clinical breakthroughs in overcoming tumor radioresistance and driving substantial progress in cancer treatment strategies.

## Glossary

NEAA: non-essential amino acid
TCA: tricarboxylic acid
GLS: glutaminase
GAC: glutaminase C
α-KG: alpha-ketoglutarate
GLUD1/GDH1: glutamate dehydrogenase 1
GSH: glutathione
RR: radiotherapy resistance
CSCs: cancer stem cells
DSBs: DNA double-strand breaks
IR: ionizing radiation
SSBs: single-strand breaks
DDR: DNA damage response
BER: base excision repair
NER: nucleotide excision repair
HR: homologous recombination
NHEJ: nonhomologous end-joining
PCa: prostate cancer
GS: glutamine synthetase
PPAT: phosphoribosyl pyrophosphate amidotransferase
PRPP: phosphoribosyl pyrophosphate
PRA: phosphoribosylamine
PFAS: phosphoribosylformylglycinamidine synthase
FGAR: N2-formyl-N1-(5-phospho-d-ribosyl)glycinamide
FGAM: 2-(formamido)-N1-(5-phospho-d-ribosyl) acetamidine
GMPS: GMP synthetase
CPS: carbamoyl phosphate synthetase
IMP: inosine monophosphate
AMP: adenosine monophosphate
GMP: guanosine monophosphate
CAD: Dihydroorotase
CP: carbamoyl phosphate
DHODH: dihydroorotate dehydrogenase
GATase: glutamine amidotransferase
ATCase: spartate transcarbamylase
DHOase: dihydroorotase
C-Asp: carbamoyl aspartate
DHOA: dihydroorotate
OA: orotate
UTP: uridine triphosphate
UMPS: uridine 5′-monophosphate synthase
UMP: uridine monophosphate
CTP: cytidine triphosphate
CTPS: CTP synthase
dNTPs: deoxynucleoside triphosphates
RNR: ribonucleotide reductase
UCK: uridine-cytidine kinase
PTMs: posttranslational modifications
O-GlcNAcylation: O-linked β-N-acetylglucosamine modification
O-GlcNAc: O-linked β-N-acetylglucosamine
OGT: O-linked N-acetyl-glucosaminyltransferase
OGA: O-linked N-acetyl β-D-glucosaminidase
HBP: hexosamine biosynthetic pathway
UDP-GlcNAc: uridine 5′-diphosphate(UDP)-N-acetylglucosamine
GFAT: glutamine-fructose-6-phosphatase aminotransferase
Fru-6-P: fructose-6-phosphate
GlcN-6-P: glucosamine-6-phosphate
MDC1: mediator of DNA damage checkpoint 1
ATM: ataxia telangiectasia mutated
TNBC: triple-negative breast cancer
GBM: glioblastoma
CRC: colorectal cancer
NSCLC: non-small-cell lung cancer
**NAD** ^**+**^: nicotinamide adenine dinucleotide
PRPS1: phosphoribosyl pyrophosphate synthetase one
PARylation: poly-ADP ribosylation
PARPs: poly (ADP-ribose) polymerases
PARP-1: poly (ADP-ribose) polymerase 1
ALC1: amplified in liver cancer 1
CHD1L: chromodomain-helicase-DNA-binding protein 1-like
CHD3: chromodomain helicase DNA binding protein 3
CHD4: chromodomain helicase DNA-binding protein 4
HPF1: histone PARylation factor 1
CTCF: CCCTC-binding factor
DNA-PK: DNA-dependent protein kinase
NONO: non-POU domain–containing octamer-binding
DNA-PKcs: DNA-PK catalytic subunit
And-1: acidic nucleoplasmic DNA-binding protein 1
NBS1: nijmegen breakage syndrome protein 1
PARylation: poly-ADP-ribosylation
PH: Preiss Handler Pathway
NAMPT: nicotinamide phosphoribosyltransferase
NMN: nicotinamide mononucleotide
HMGB1: high-mobility group box 1
ROS: reactive oxygen species
NADPH: nicotinamide adenine dinucleotide phosphate
GCLC: glutamate‐cysteine ligase catalytic subunit
SLC7A11: solute carrier family 7 member 11
GSSG: glutathione disulfide
GR: glutathione reductase
GDH1: glutamate dehydrogenase 1
GOT1: aspartate transaminase
MDH1: malate dehydrogenase
ME1: malic enzyme 1
PPP: pentose phosphate pathway
GPX4: glutathione peroxidase 4
PL-OOHs: phospholipid hydroperoxides
ASCT2: alanine-serine-cysteine transporter 2
MMS: methyl methanesulfonate
PDT: photodynamic therapy
GPNA: γ-L-glutamyl-p-nitroanilide
AOA: aminooxyacetate
Ce6: photosensitizers chlorin e6
PPa: pyropheophorbide a
PDT: photodynamic therapy
GDH: glutamate dehydrogenase
EGCG: epigallocatechin gallate
DON: 6-Diazo-5-oxo-L-norleucine.
